# Perspective Taking Ability in Psychologically Maltreated Children: A Protective Factor in Peer Social Adjustment

**DOI:** 10.3389/fpsyg.2022.816514

**Published:** 2022-03-03

**Authors:** Ada Cigala, Arianna Mori

**Affiliations:** Department of Humanities, Social Sciences and Cultural Industries (DUSIC), University of Parma, Parma, Italy

**Keywords:** perspective taking, social adjustment, prosocial behaviors, aggressiveness, maltreatment, preschoolers

## Abstract

Perspective taking is conceptualized as a multidimensional construct characterized by three components: cognitive, affective, and visual. The experience of psychological maltreatment impairs the child’s emotional competence; in particular, maltreated children present difficulty in understanding and regulating emotions and in social understanding ability. In addition, the literature contains several contributions that highlight maladaptive behaviors of children with a history of maltreatment in peer interactions in the school context. Perspective taking ability has rarely been studied in maltreated children and the existing studies have produced different and often conflicting results that require further insights. On the grounds of these premises, the main objective of the present research is to investigate perspective taking ability in preschool children from maltreating and non-maltreating family contexts and its role in social adjustment, in terms of prosocial and aggressive behavior toward peers inside the kindergarten. A second objective is to verify the effectiveness of a training aimed to promote perspective taking ability in victims of psychological maltreatment. This research, organized into two separate studies, involved 249 preschool children: 206 children from non-maltreating family contexts and 43 brought up in psychologically maltreating families. Perspective taking was measured *via* the administration of several tests, and prosocial behavior and aggressiveness were observed *via* non-participant observations in the school context. The training involved maltreated children in small-group meetings based on familiar and appealing activities within the mother–child community. The overall results show that children’s perspective taking ability, in particular the affective perspective taking, contributed to social adjustment. In fact, greater affective perspective taking ability was correlated to a higher frequency of prosocial behaviors toward peers and minor frequency of aggressiveness. Finally, the results of the training (pre/post-test comparison) showed an increase in perspective taking, especially in the affective dimension, and a consequent increase in prosocial behaviors and a decrease in aggressive ones. Therefore, the affective perspective taking ability seems to represent a very significant protective factor, which should be focused and strengthened in order to improve the social adaptation of preschool children who are victims of psychological abuse.

## Introduction

The construct of social–emotional competence refers to a set of interrelated skills that are learned in social interactions from the earliest years of life and that are progressively developed in these interactions and which enable one to be *emotional effective* in everyday social exchanges (Saarni et al., 2007). Skills of social–emotional competence range from recognizing emotions in oneself and in others, to feeling empathy and emotional participation in the affairs of others, expressing emotions according to the performance rules of the culture they belong to, knowing the causes that provoke them, being able to name them using an appropriate emotional vocabulary and being able to self-regulate (Tucker et al., 2017; Murano et al., 2020).

One core social–emotional skill is perspective taking. Some authors consider perspective taking as a multidimensional construct characterized by three components: cognitive, affective, and visual. *Cognitive perspective taking* refers to the understanding of others thoughts and intentions (Baron-Cohen, 2001; Eisenberg et al., 2001), the *visual* one is identified as the ability to make inferences about how an object is seen by a person occupying a different position (Vogeley and Fink, 2003; Moll and Tomasello, 2006; Moll and Meltzoff, 2011; Frick et al., 2014) and the *affective* one as the ability to understand others’ emotional states (Harwood and Farrar, 2006; Hinnant and O’Brien, 2007; Fireman and Kose, 2010; Sette et al., 2015).

Research into these issues has revealed that perspective taking could influence children’s social adjustment. In fact, it seems that perspective taking is related to a constellation of social and relational skills that underlie positive interactions with the outside world: the ability to read and decode social cues, to resolve conflict, to adjust adequately one’s own emotions to the context and to manifest prosocial and altruistic behaviors (Eisenberg et al., 2003). Research findings indicate that people more skilled at perspective taking are less likely to stereotype others (Galinsky and Moskowitz, 2000), respond less aggressively when provoked (Richardson et al., 1998), and develop more positive relationships with those who hold beliefs that differ from their own (Galinsky and Moskowitz, 2000; Gehlbach et al., 2015). Other research (Gal Endres, 2003) has shown that the development of perspective taking is significantly related to social competences and that “good perspective takers” are also considered more socially competent by their teachers (Lalonde and Chandler, 1995), and are more accepted by friends (Klin et al., 2000; Fitzgerald et al., 2003).

Based on the evidence of these positive influences of perspective taking on children’s social adjustment, many authors have investigated the possibility of educating children to take on the perspective of others through specific training and then measuring its effectiveness. Results obtained from research articles and literature reviews (Hofmann et al., 2016; Mori and Cigala, 2016) show that targeted intervention procedures can effectively increase perspective taking, both in cognitive (Tsuji, 2020), visual and affective dimensions (Ornaghi et al., 2015), whether the focus is on enhancing one dimension at a time or on perspective taking in its multidimensional nature (Cigala et al., 2015; Mori and Cigala, 2016). Moreover, Mori and Cigala (2016), have shown that, in general, the implemented trainings refer to three main analytical perspectives: the cognitive approach (Theory of Mind) the behaviorist approach (Relational Frame Theory) and finally, the socio-constructionist approach. These approaches, although differing in the dimension of perspective taking investigated and the methodologies used, have made it possible to demonstrate that it is indeed possible to help preschool children to understand other people’s points of view.

Given the adaptive nature of perspective taking and, in particular, its contribution to the children’s social adjustment, some scholars have analyzed this ability in children with a history of maltreatment. Psychological maltreatment in general and its subcategories of neglect and assisted violence can result in short-term effects and long-term physical and psychological symptoms that usually characterize a child’s entire development (Margolin and Gordis, 2004; Fantuzzo and Fusco, 2007; Pears et al., 2010; Moreno-Manso et al., 2017). There is empirical evidence that maltreatment impairs the child’s emotional competence in particular, maltreated children present difficulty in understanding and regulating emotions and in social understanding ability (Cicchetti et al., 2003; Pears and Fisher, 2005; Shipman et al., 2007; Luke and Banerjee, 2013; Cigala and Mori, 2016a,b). In addition, the literature contains several contributions that highlight maladaptive behaviors of children with a history of maltreatment in peer interactions in the school context. In particular, studies have shown a difficulty for maltreated children to implement prosocial and empathic behaviors, a greater tendency to behave aggressively and impulsively, externalizing behavioral disorders, and maladaptive management of peer relationships (Fantuzzo et al., 1991; Martin and Clements, 2002; Holmes, 2013; Moreno-Manso et al., 2017; Dickerson et al., 2019; Thibodeau et al., 2019).

The socio-constructive perspective attributes a fundamental role to the family context and in particular to the type of family relationships in the development of perspective taking skills (Cicchetti and Lynch, 1995). Growing up in a maltreating family context, often poor in supporting elements, empathy, and appropriate educational practices, essential to developing the ability to understand the feelings, thoughts, and perspectives of others could represent a threat for the development of appropriate perspective taking abilities (Macfie et al., 2001; Cicchetti and Toth, 2005; Burack et al., 2006). In addition, inadequate practices of emotional socialization could be a barrier to the construction in the family of a space of sharing and explication of emotions within which children can learn to attribute meanings to their own and other emotions (Meins et al., 2002, 2003; Cicchetti et al., 2003; Cigala and Mori, 2014). Support, affection, empathic modeling, conversations about other people’s inner states are important variables in the development of the ability to understand others’ feelings and perspectives, and to have a coherent sense of self (Cornell et al., 2017; Ornaghi et al., 2020).

Among the components of socio-emotional competence investigated in relation to maltreatment situations, perspective taking ability rarely appears, and the existing studies have produced different and often conflicting results that require further insights. In particular, a study focused on the cognitive dimension of perspective taking, demonstrating poorer abilities in solving false beliefs tasks in maltreated children compared to non-maltreated ones. This study considered children abused physically, sexually, and emotionally from 4 to 8 years of age (Cicchetti et al., 2003). Other authors (Pears and Fisher, 2005) analyzed the ability of perspective taking by separating the affective dimension from the visual-cognitive one in preschool children victims of different types of maltreatment (e.g., physical, sexual, emotional abuse, and neglect), inserted in family planning programs. The results showed that the maltreatment conditions are significantly associated with a poor understanding of all basic emotions (e.g., happiness, fear, sadness, and anger) and difficulties in solving visual and cognitive decentralization tasks. From a literature review (Luke and Banerjee, 2013) a lack of agreement has emerged in the literature with respect to the consequences of maltreatment on perspective taking skills in school children. In particular, some studies have reported poorer perspective taking abilities in maltreated children than in non-maltreated peers in tasks when they were asked to retell a story that had just been heard from another person’s point of view (Barahal et al., 1981; Burack et al., 2006).

However, other research did not reveal significant differences in performance in perspective taking tasks between physically abused children (Walker and Downey, 1990) and the victims of multiple forms of abuse (physical, sexual, emotional, and neglect; Lazaro and Lopez, 2010) and non-maltreated peers. Finally, from research that adopted a multidimensional approach on perspective taking (Cigala and Mori, 2014) no significant differences emerged with regard to the visual and cognitive dimension of perspective taking between maltreated and non-maltreated children. The only differences emerged in the affective component, in which maltreated children showed worse performances, demonstrating their lower ability to understand emotional expressions and emotional situations and to predict their own emotion of sadness.

On the grounds of these premises, we consider that there is a need for further studies that investigate perspective taking ability in order to better understand the relationships between children’s perspective taking ability and social adjustment in peer interactions, both in the presence of maltreatment and in typically developing groups. In particular, starting from the analysis of the cited recent literature (Luke and Banerjee, 2013; Tejada and Linder, 2020) the need emerges to focus future research on preschool age that has hardly at all been investigated. In fact, in the field of maltreatment, many more studies on the relationship between emotional competence and prosocial behavior have been carried out on children of school age. The decision to focus this study on the preschool age group was based on the evidence that it represents a particularly salient phase in the development of perspective taking. It is, in fact, from about 3 to 6 years of age that the ability to understand points of view other than one’s own emerges, develops, and, above all, consolidates in children. Finally, the preschool age is more significant at the level of prevention and intervention: In this age period, the maladaptive “vicious circle” between the family context and adaptation with peers is not yet so consolidated and structured. It is, therefore, very interesting to carry out investigations at an increasingly early age in order to gather evidence that can enable the implementation of paths to promote perspective taking in children (Self-Brown et al., 2012; Toth and Cicchetti, 2013; Moreno-Manso et al., 2017).

### The Current Contribution

Based on the previous premises, the main aim of the present research was to investigate the perspective taking ability in preschoolers belonging to maltreating and non-maltreating family contexts and the influence that perspective taking ability could have on social adjustment in terms of prosocial and aggressive behaviors toward peers inside the kindergarten. In particular, two different studies were implemented.

#### Study I Had Two Main Objectives

- To compare maltreated and non-maltreated children with respect to perspective taking and adaptive functioning in the school context, expressed in terms of prosocial behaviors and aggressiveness, also controlling the influence of other variables: gender, siblings, and age. We hypothesized to detect differences between the two groups, with regard to the affective dimension of perspective taking, and in particular, we expected to find better affective decentralization skills in children belonging to a normative family context (Cigala and Mori, 2014). As regards social adjustment, we expected to detect a higher frequency of aggressive behaviors among maltreated children (Fantuzzo et al., 1991; Martin and Clements, 2002; Holmes, 2013). In line with the literature, we did not expect to find gender differences in perspective taking ability but more aggressive behaviors and prosocial behaviors in males than in females (Rose and Rudolph, 2006). Finally, we expected that having siblings could promote the perspective taking ability akin to what occurs with other similar social skills (Perner et al., 1994; Ruffman et al., 1998).
- To assess whether perspective taking ability could play a role in social adaptation, expressed in terms of prosocial behaviors and aggressiveness with peers in a group of children with a history of psychological maltreatment and in a group belonging to non-maltreating families. We expected that children’s social adjustment expressed in terms of prosocial peer behavior could be positively related to the perspective taking ability of children in both groups. On the other hand, we expected that children’s social maladjustment expressed in terms of aggressive peer behavior could be negatively correlated to children’s perspective taking abilities (Carlo, 2005; Dodge et al., 2006; Cigala et al., 2015; Mori and Cigala, 2019).

#### Study II Had Two Main Objectives

- To verify the efficacy of a specific group training in promoting perspective taking ability in maltreating children.
- To verify whether the enhancement of the visual, affective, and cognitive perspective taking ability in the group of maltreated children following a specific training could improve social adjustment in terms of prosocial and aggressive behaviors among peers in the school context. The hypothesis was that after the training, there would be an increase in perspective taking abilities (visual, cognitive, and affective); moreover, the advancement of perspective taking was associated with an increased frequency of prosocial behavior and a reduction in aggressiveness (Cigala et al., 2015; Mori and Cigala, 2019).

## Study I

### Materials and Methods

#### Participants

The group of participants comprised 249 preschoolers: 206 children belonging to non-maltreated families and 43 children, victims of psychological abuse. The 206 non-maltreated preschoolers (without referral by social services) are aged between 45 and 65 months (*M* = 54.72, *SD* = 4.61), 104 males (*M* = 54.65, *SD* = 4.49) and 102 females (*M* = 54.78, *SD* = 4.74); 54 children are an only child and 152 have at least one sibling. Children attended 10 classes in 8 kindergartens in northern Italy and belonged to middle socioeconomic status families. For the formation of the maltreated children’s group, the following inclusion criteria were established: (1) Age between 3 and 5 years; (2) Absence of clinical diagnosis; (3) Presence of psychological abuse (neglect/assisted violence); (4) Knowledge of Italian (both comprehension and production). The presence of psychological maltreatment was ascertained with interviews with the directors of the communities in which the research was conducted (all having professional profiles as psychologists).

The 43 preschool victims of psychological abuse are aged between 36 and 68 months (*M* = 53.07, *SD* = 9.41), 21 males (*M* = 51.19, *SD* = 8.87) and 22 females (*M* = 54.86, *SD* = 9.77); 25 children are an only child and 18 have at least one sibling. Children residing, together with their mothers, in 15 mother–child communities are located in 9 Northern Italian provinces. All the children were Italian native speakers and had Italian nationality, although 18 came from families of foreign origin (10 from North Africa, 4 from Central Africa, and 4 from Eastern Europe).

Of the 15 considered communities, six were of a therapeutic nature, in which mothers were on a rehabilitation path to recover from drugs, alcohol, and medicines and 9 were so-called “welcoming communities,” in which the hosted women presented a variety of problems (e.g., social malaise, poverty, application for political asylum, and conflicting separations). Specifically, 17 children lived in therapeutic communities and 26 in host communities. The average residence time within the communities was 18.91 months (*SD* = 13.945; Range: 1–52 months). Only 25 children had sporadic contacts with their fathers (once or twice a month) usually during supervised meetings in the presence of educators of the community, often inside the prison where the father was being detained.

#### Design and Procedure

This study is part of a larger project that involves the collaboration between University, the School Institution, and a *Mother–Child Communities* network in northern Italy. Before launching the study, a preliminary phase was implemented during which the kindergartens were contacted, through the managers and coordinators of the Educational Services of the various municipalities or the school directors. After obtaining initial consent to participate, the explanatory research project was sent to the school containing a detailed description of the objectives, methods, and timeframe for carrying out the research. The next step was to present the project first to the teachers and then to the parents, who were given the informed consent form. These meetings proved to be particularly useful as they gave everyone the opportunity to listen, reflect, make and answer questions, raise doubts and uncertainties that could have been an obstacle for the continuation of the research.

For the children belonging to maltreated families, the preliminary phase consisted in contacting therapeutic/welcoming communities (20 communities), in some cases with the prior consent of the Social Services, in others through direct agreements with the managers of each organization. Fifteen communities (6 therapeutic and 9 welcoming) accepted to participate in the research and received the research project. The next phase involved presenting the project first to the directors and educators of the communities and then to the mothers, who were given the specific document for their informed consent.

#### Instruments

##### Socio-Emotional Competence: Perspective Taking

For each child, affective, cognitive, and visual perspective taking were assessed. Three tasks were proposed to each child in order to detect each component of perspective taking. The perspective taking instruments were administered individually in three 20–25-min meetings in a quiet room of the kindergarten or the community. These tasks were pleasant, non-invasive, and were designed to not evoke emotionally distressing situations for the children (Table 1). The administration of the task battery was conducted by a trained research assistant, who was blind to the specific aims and hypotheses of the study.

**Table 1:** Perspective taking assessment tasks (Study I).

Affective PT	Cognitive PT	Visual PT
TEC (Pons and Harris, 2000)	Sally and Anne (Wimmer and Perner, 1983)	The grub sleeping on a pillow (adapted from Flavell et al., 1981)
Emotional decentralization task (Harwood and Farrar, 2006)	Nesquik box with pencils (re-adapted from Perner et al., 1987)	The land before time (adapted from Hughes and Donaldson, 1979)
Rainbow and his friends (adapted from Donovan, 2002)	Rubber-walnut (adapted from Flavell, 1993)	The crocodile (adapted from Flavell, 1966)

*Cognitive perspective taking* was assessed by classical false belief tasks (*unexpected location and unexpected content*; Wimmer and Perner, 1983; Baron-Cohen et al., 1985; Perner et al., 1987) and the appearance–reality distinction tasks (*deceptive object*; Flavell et al., 1986) whose assumption is the child’s ability to attribute different thoughts and beliefs to other people.

Three types of tasks were proposed for the analysis of *visual perspective taking*. Two of them are *visual-perceptual tasks* (Flavell et al., 1981; Mori and Cigala, 2019): children were shown two tables featuring the same sketch/image on both sides, but with a different detail; the challenge was to consider both points of view. The third test is represented by a modified and adapted version of the *“policeman task”* (Hughes and Donaldson, 1979; Mori and Cigala, 2019) consisting of a three-dimensional model in which the child’s task is to simulate the game of hide-and-seek, placing a character in such a way that the other character cannot see. The scene is presented so that the child’s point of view does not coincide with the characters’ point of view.

For the analysis of *affective perspective taking* the Italian version of the TEC (*Test of Emotion Comprehension*; Pons and Harris, 2000; Albanese and Molina, 2008) was first proposed to the children. Secondly, an adapted version of the *emotional decentralization task* (Harwood and Farrar, 2006) was proposed, in which the children were presented 12 short stories involving themselves and a friend. After each story, the researcher asked how the child and his/her friend would feel in such a situation. Finally, each child was told an illustrated story, specifically created for the recognition of the four basic emotions (i.e., happiness, fear, anger, and sadness) and asked to recognize the emotion felt by the involved characters.

##### Social Adjustment: Prosocial Behavior and Aggressiveness

*Prosocial behavior* and *aggressiveness* were investigated through direct non-participant observations by means of an observation grid, created on the basis of prosocial behaviors assessment code systems (D’Odorico et al., 2000; Roche Olivar, 2002; Bergin et al., 2003; Cigala et al., 2015; Mori and Cigala, 2019). Observations were conducted in the kindergarten attended by children in three different 45-min sessions on different days during lunch and in moments of free play both before and after lunch. Target behaviors (more than fifty) were operationalized into 4 behavioral categories: *helping* (e.g., to help a friend in need and to help a friend wash their hands), *consoling/supporting* (e.g., to get close to a sad friend and to give a toy to a sad friend) and *sharing* (e.g., to share toys or materials with friends and to tell personal experiences with friends) that we considered as prosocial behaviors, and *aggressiveness* (e.g., physically assaulting a peer by pushing, biting or scratching, and taking over other children’s games even if they complain). The observer, blind to the hypotheses and previously trained, noted in the grid every time a target behavior was performed by the observed child. Then the behaviors were summed, so as to evaluate the frequency of emission of each of the aforementioned behavior categories. This coding grid had already been used in previous research (Cigala et al., 2015), and Cronbach’s alpha score for the reliability of the internal consistency of the coding grid is 0.69 for each behavior separately.

### Results

#### Score Coding

Before the analysis of the results, the scores obtained by the children in the individual perspective taking tasks and in the observation sessions had to be recoded. The recoding procedure was based on the one already used in other studies (see Mori and Cigala, 2019). In order to have a single perspective taking indicator for each dimension, first, three new scores for each dimension (*affective, cognitive,* and *visual PT*) were calculated according to following formula:


$$\left[\begin{array}{c} \mathrm{PT}\;\mathrm{index}=\left(\mathrm{score}\;\mathrm{on}\;\mathrm{task}1/\max\;\mathrm{score}\;\mathrm{on}\;\mathrm{task}\;1\right)+\\ \left(\mathrm{score}\;\mathrm{on}\;\mathrm{task}2/\max\;\mathrm{score}\;\mathrm{on}\;\mathrm{task 2}\right)+\\ {\left(\mathrm{score}\;\mathrm{on}\;\mathrm{task}3/\max\;\mathrm{score}\;\mathrm{on}\;\mathrm{task 3}\right)}^{*}1/3 \end{array}\right]$$

Then the values were multiplied by 100 in order to increase readability. In this way, we obtained three new scores ranging from 0 (poorer performance) to 100 (best performance): visual PT, affective PT, and cognitive PT. Moreover, a global Index of perspective taking was computed by averaging the three scores described (*totalPT*).

In a previous study, in order to evaluate the extent to which the different test would measure similar construct, a confirmatory factor analysis was performed for pre-test and post-test measures separately (see Mori and Cigala, 2019). More precisely, a model with three latent factors (i.e., affective, cognitive, and visual PT) each measured by three indicators was tested using maximum likelihood estimation with robust standard error. For the pre-test, the results indicated a good fit of the three-factor model, v2 (23) = 8.13, *p* = 0.99, CFI = 1.00, RMSEA = 0.00 [90% CI = 0.00–0.00], *p* = 0.99, SRMR = 0.022. Moreover, all the observed variables were significantly measured by the intended latent factor (ps ≤ 0.05). Factors core determinacy was 0.81 for affective, 0.77 for cognitive, and 0.93 for visual dimension of PT. Similar results were obtained for post-test measures. Indeed, goodness of fit was acceptable, v2 (23) = 33.02, *p* = 0.08, CFI = 0.966, RMSEA = 0.046 [90% CI = 0.000–0.079], *p* = 0.54, SRMR = 0.044, and all observed variables were significantly represented by the intended latent dimension (ps < 0.01). Factor score determinacy was 0.96 for affective, 0.84 for cognitive, and 0.84 for visual dimension of PT. Finally, an indicator of prosocial behavior was also computed by summing the frequency of every single behavioral category: *helping*, *consoling/supporting,* and *sharing* (*Prosocial total*). An indicator of aggressiveness was also computed by summing the frequency of every single aggressive behavioral (*aggressiveness*).

#### Analysis and Results

In relation to the first objective of the study I, the two groups of maltreated and non-maltreated children were compared by means of a multivaried analysis with respect to perspective taking and adaptive functioning, expressed in terms of prosocial behaviors and aggressiveness. A multivariate analysis was conducted with the presence of *maltreatment* as independent variable; *age, gender,* and the presence of *siblings* as covariates; *affective, cognitive,* and *visual perspective taking*, *prosocial* and *aggressive behaviors* as dependent variables. From this analysis a significant effect of maltreatment on *affective perspective taking* [*F*_(1,244)_ = 28.12, *p* < 0.001], *prosocial behaviors* [*F*_(1,244)_ = 9.98, *p* = 0.002], and *aggressive behaviors* with peers [*F*_(1,244)_ = 27.55, *p* < 0.001] emerged. The analysis of the mean values (Table 2) showed that the group of maltreated children was characterized by a lower affective perspective taking, a lower frequency of prosocial behaviors, and a greater frequency of aggressive behavior toward peers.

**Table 2:** Perspective taking, prosocial, and aggressive behaviors for the presence of maltreatment: descriptive statistics.

	Non-maltreated (*N* = 206)		Maltreated (*N* = 43)		Total (*N* = 249)	
	*M*	*SD*	*M*	*SD*	*M*	*SD*
Affective PT	58.43	11.98	47.62	14.62	56.56	13.10
Cognitive PT	53.51	22.96	55.55	26.45	53.86	23.55
Visual PT	69.66	28.90	67.05	35.73	69.21	30.12
Total PT	60.53	15.35	57.20	13.81	59.96	15.12
Prosocial total	13.18	4.95	10.42	5.34	12.71	5.11
Aggressiveness	1.81	3.54	5.77	6.34	2.49	4.41

With respect to the second objective, two linear regression models were conducted in order to verify the predictor variables of prosocial and aggressive behavior among peers in school contexts.

In particular, in the first model, the *total prosocial behavior* (derived from the sum of the *help, consoling,* and *sharing* behaviors) was considered as dependent variable, while the following were included as predictors: *visual, affective, and cognitive perspective taking*, *age* (younger/older based on the average value), *gender, sibling* (only child/at least one sibling) and the presence of maltreatment (*maltreatment*/*non-maltreatment*).

The zero-order correlation between *perspective taking* and *prosocial behaviors* (Table 3) pointed out that children’s ability in *affective perspective taking* (*p* < 0.01) and *cognitive perspective taking* (*p* < 0.05) are positively correlated with the frequency of *prosocial behaviors*.

**Table 3:** Zero-order correlation between perspective taking, prosocial behaviors, and aggressiveness.

S. No.		1	2	3	4	5
1.	Affective PT	–	0.251^**^	0.281^**^	0.231^**^	−0.347^**^
2.	Cognitive PT		–	0.109	0.145^*^	0.003
3.	Visual PT			–	0.076	−0.019
4.	Prosocial total				–	−0.161^*^
5.	Aggressiveness					–

* *p* < 0.05, *N* = 249.

** p < 0.01, *N* = 249.

The regression model was significant [*F*_(7,241)_ = 3.23, *p* = 0.003, *R*^2^ = 0.09; *VIF = range* 1.04–1.20*; VIF Tolerance* > 0.2]. The significant predictors were: *affective perspective taking* (*b* = 0.60, *SE* = 0.03, *p* = 0.032), and the presence of *maltreatment* (*b* = −2.11, *SE* = 0.92, *p* = 0.022; Table 4).

**Table 4:** Linear Regression Model I: prosocial behaviors.

	*b*	*SE*	*Beta*	*p*
Maltreatment (vs. non-maltreatment)	−2.11	0.92	−0.16	0.02
Gender (0 = male)	−0.10	0.64	−0.01	0.88
Age	−0.27	0.65	−0.03	0.68
Siblings	0.21	0.71	0.02	0.77
Affective PT	0.06	0.03	0.16	0.03
Cognitive PT	0.03	0.01	0.12	0.08
Visual PT	0.00	0.01	0.02	0.82

In particular, the descriptive statistics (Table 2) showed that the presence of *maltreatment* is associated with lower levels of *prosocial behaviors* toward peers and the correlations (Table 3) pointed out that children’s ability in *affective perspective taking* is positively correlated with the frequency of *prosocial behaviors*.

In the second model of regression analysis, the *aggressive behaviors* were included as dependent variable, while the predictors variable were the same as in the previous model.

The zero-order correlation between *perspective taking* and *aggressive behaviors* (Table 3) pointed out that aggressive behavior is inversely correlated with *affective perspective taking*.

The regression model (Table 5) was significant [*F*_(7,241)_ = 9.77, *p* < 0.001, *R*^2^ = 0.22; *VIF = range* 1.04–1.20*; VIF Tolerance* > 0.2]. The significant predictors were: *affective perspective taking* (*b* = −0.09, *SE* = 0.02, *p* < 0.001), the presence of *maltreatment* (*b* = 2.65, *SE* = 0.72, *p* < 0.001) and the *gender* (*b* = −1.32, *SE* = 0.51, *p* = 0.010).

**Table 5:** Linear Regression Model II: aggressive behaviors.

	*b*	*SE*	*Beta*	*p*
Maltreatment (vs. non-maltreatment)	2.65	0.73	0.23	0.001
Gender (0 = male)	−1.32	0.51	−0.15	0.01
Age	−0.36	0.52	−0.04	0.48
Siblings	−0.89	0.57	−0.09	0.12
Affective PT	−0.09	0.02	−0.28	0.00
Cognitive PT	0.01	0.01	0.07	0.25
Visual PT	0.01	0.01	0.07	0.23

In particular, the presence of *maltreatment* (*M* = 5.77, *SD* = 6.34 vs. *M* = 1.81, *SD* = 3.54) and male *gender* (*M* = 3.30, *SD* = 5.17 vs. *M* = 1.68 *SD* = 3.29) are associated with a higher frequency of aggressive behaviors among peers (Table 2), while *affective perspective taking* is inversely correlated with aggressive behavior (Table 3).

## Study II

### Materials and Methods

#### Participants

The group of participants, consisting of 43 children victims of psychological abuse, corresponds to that of study I, whose characteristics have been described above. None of the children had previously taken part in similar research.

#### Design, Instrument, and Training

The study was conducted with a quasi-experimental design composed of three phases: pre-test (T1), training (T2), and post-test (T3). The aim of the *pre-test* (T1) was to assess the child’s perspective taking abilities, prosocial behaviors, and aggressiveness toward peers, and it corresponds to the data collection of the previous study.

The *training phase* (*T2*) had the aim of promoting the children’s ability to take others’ point of view through various activities according to a protocol created *ad hoc* that have been shown to be effective in the literature (Cigala et al., 2015; Mori and Cigala, 2019). The training, described in all its parts in previously studies (Cigala et al., 2015; Mori and Cigala, 2019, 2021), consists of nine sessions (three for each perspective taking dimension) with a three-weekly frequency, lasting 45 min each. In these sessions, the children were proposed some activities, such as reading stories, reflection/discussion, dramatization, drawing, and empirical exercises of decentralization, in order to enhance their ability to assume others’ visual, affective, and cognitive perspective. The activities were proposed to small groups of children (about 4–5 children for each group) within the community. As described in previous articles (Mori and Cigala, 2019), the adult’s role in this training is to guide the activities, supporting a mutual exchange among the children allowing each of them, without pressure, to express and share their thoughts with their classmates. The adult also provides explanatory feedback, in order to help each member of the group take on other perspectives. Precisely, the trainer in this path had two-fold role: a. to put forward situations and stimuli to promote the children’s awareness of their own point of view (i.e., visual, cognitive, and affective) and the awareness of the existence of points of view different from their own; b. to highlight the coexistence of different looks and perspectives creating in the various meetings a perspective taking space, in which different perspectives can coexist and be explained. The training had already been used with children of this age with typical development and the measure treatment fidelity inter-rater reliability calculated across the sessions was 80% (Mori and Cigala, 2019).

In the *post-test* phase (T3), followed the training, perspective taking abilities, prosocial behaviors, and aggressiveness toward peers were assessed again, in three sessions lasting 20–25 min each, by administering different but similar versions of the same instruments as in the pre-test (Table 6). For the assessment of prosocial and aggressive behaviors, the same observational procedure was used as for the pre-test.

**Table 6:** Perspective taking assessment tasks: pre-test and post-test (Study II).

	Affective PT	Cognitive PT	Visual PT
PRE-TEST	TEC (Pons and Harris, 2000)	Sally and Anne (Wimmer and Perner, 1983)	The grub sleeping on a pillow (adapted from Flavell et al., 1981)
	Emotional decentralization task (Harwood and Farrar, 2006)	Nesquik box with pencils (re-adapted from Perner et al., 1987)	The land before time (adapted from Hughes and Donaldson, 1979)
	Rainbow and his friends (adapted from Donovan, 2002)	Rubber-walnut (adapted from Flavell, 1993)	The crocodile (adapted from Flavell, 1966)
POST-TEST	TEC (Pons and Harris, 2000)	The mice “Stefano e Alberto” (Liverta Sempio et al., 2005)	The turtle sleeping on a pillow (Flavell et al., 1981)
	Emotional decentralization task (Harwood and Farrar, 2006)	Bottle water with candy (Perner et al., 1987)	The Lego farm (Hughes and Donaldson, 1979)
	Pingu and his friends (Von Flüe, 1990)	Syringe-Highlighter (adapted from Flavell et al., 1986)	The dog (Flavell et al., 1981)

### Results

For the evaluation of the effectiveness of the training, repeated-measure analysis with pre- and post-test within-subject variables was implemented. In these analyses, the *perspective taking* scores (affective, visual, cognitive dimensions, and total scores), *aggressive* and *prosocial behaviors* were considered as between-subject variables. The results are reported in Table 7.

**Table 7:** Descriptive statistics and values of the pre-test/post-test variables.

	*N*	Pre		Post		*F*(*1*,*42*)	*p*
		*M*	*SD*	*M*	*SD*		
Affective PT	43	47.62	14.62	63.15	13.23	48.86	<0.001
Cognitive PT	43	55.55	26.45	80.88	15.40	24.52	<0.001
Visual PT	43	67.05	35.73	62.02	29.62	0.55	0.463
Total PT	43	57.20	13.81	68.68	13.78	15.29	<0.001
Prosoc_tot	43	10.42	5.34	13.02	5.84	15.95	< 0.001
Helping	43	2.09	2.40	2.65	2.77	4.18	0.047
Consoling/encouraging	43	0.81	1.03	0.84	1.25	0.02	0.912
Sharing	43	7.51	3.67	9.53	3.71	13.18	0.001
Aggr_tot	43	5.77	6.34	3.81	4.66	4.24	0.048

The training improved the perspective taking ability of maltreated preschoolers, in particular the *affective* and *cognitive* dimensions. No differences were detected in visual component of perspective taking before and after the training (Table 7). With respect to social adjustment, an increase in *prosocial behavior* toward peers exhibited in the school context appeared after the training and this was due mainly to improving in sharing behavior and, with a lesser extent, to helping behavior (Table 7). Similarly, the *aggressive behaviors* toward peers decreased after the training, form pre-test to post-test.

### Discussion

Overall, the data emerging from the study seems to advance our knowledge in several directions. On the one hand, they help to clarify the relationship between the social adjustment of preschool children and their perspective taking ability. On the other hand, the data contribute to understanding the development of perspective taking in children with a history of maltreatment, also concurring to integrate the evidence in the literature, which, as seen in the Introduction, at the present time appears to be inconsistent (Cicchetti et al., 2003; Pears and Fisher, 2005; Luke and Banerjee, 2013).

In particular, the first study shows that children’s social adaptation, expressed in terms of prosocial behavior in the school context toward peers, is significantly influenced by the ability of affective perspective taking. It emerges, then, that children’s ability to recognize and understand the emotional states of others, identifying both positive and negative emotions (especially sadness and anger), represents a competence that can promote prosocial behaviors of help, comfort, and sharing among peers in the school context.

It is interesting to note that the other dimensions of perspective taking, particularly the visual one, seem to be less related to social adjustment. The data on cognitive perspective taking, that is, children’s ability to understand another person’s thoughts in a given situation, are worth noting. Although this component of perspective taking is significantly correlated with prosocial behavior, it is close to statistical significance in the regression model as a predictor, although it does not reach it. This finding would seem partially incongruent with several previous studies that have shown a relationship between theory of mind and prosocial behavior (Imuta et al., 2016). However, it is believed that age probably plays an important role in explaining these results. In fact, the Theory of Mind at preschool age is at an early stage of acquisition; it is still immature level of development probably explains the difficulty of transferring this understanding to the level of behavior in different contexts, such as school. Otherwise, the understanding of other people’s emotions begins to develop even before the age of 3, this probably allows affective perspective taking to be more transferable to children’s behavior (Fernández-Sánchez et al., 2014).

The data also show that the ability of affective perspective taking is associated with the aggressive behaviors toward peers. This means that children who show a lower capacity of emotional decentralization (i.e., to grasp the emotional states of another person in a specific situation) tend to behave aggressively toward peers in the school environment. It is likely that, as other studies have shown (Grolnick et al., 1999; Vallotton and Ayoub, 2010), a child’s adequate understanding of a peer’s emotional state enables them to regulate their own emotional experience of anger in certain situations and specifically to regulate behavioral manifestations or impulses to act accordingly, in order to be better adapted to the context. Affective perspective taking thus represents a key competence related to the development of good social adaptation in peer relationships in the school context, both in terms of implementing prosocial behaviors of help, comfort, and sharing and in terms of mitigating aggressive behavior.

Finally, the analyses of the first study show that there are other variables that seem to contribute to the social adaptation of preschool children, such as history of maltreatment and gender. With regard to the latter variable, gender does not seem to play a significant role in predicting prosocial behavior, but rather aggressive behavior, as several studies have shown and as had been hypothesized, connotes males more than females (Rose and Rudolph, 2006; Hastings et al., 2007). This finding seems to be congruent with studies on gender stereotyping in emotional socialization, which show, for example, that caregivers are more likely to tolerate negative reactive emotions, such as anger, in male children than in female children (Eaton, 1983; DiDonato et al., 2012).

In contrast to other studies (Hughes et al., 2018), there is no significant role of the presence of siblings in the development of prosocial or aggressive behavior. These data could also be affected by the fact that in the group of children with a history of maltreatment, the sibling variable differs from that of non-maltreated children, since the presence of siblings does not always translate into a real and daily relationship with them.

In the first study, regression analysis models show that the presence of psychological maltreatment in the children’s history is also related to a lower adaptive functioning in relationships with peers at school. In particular, maltreated children are less able to enact prosocial behaviors and are more prone to aggressive behavior. This finding supports previous studies that have identified the maladaptive functioning of these children in the school context (Cicchetti, 2013; Manly et al., 2013). The interesting aspect of the results of this study is that maltreatment, albeit having a significant role, does not turn out to be the only predictor of social functioning, but, as we have seen, in both regression models it turns out to be associated with the ability of affective perspective taking. This fact generates several reflections on the important role of affective perspective taking as a protective factor, with respect to the possibilities of social adaptation of all children and, in particular, of those with a history of maltreatment.

It is from these results and the emergence of affective perspective taking as a possible protective factor in maltreatment situations that the need arises to further investigate the developmental characteristics of perspective taking in the group of maltreated children. In this regard, the analyses have shown that it is the dimension of affective perspective taking, and not so much the cognitive and visual dimension, that appears to be more critical in the development of maltreated children compared to non-maltreated ones. This difficulty, linked in particular to the level of emotional understanding, which has already been documented in the literature by several studies (Cigala and Mori, 2016b), can be explained from different perspectives. According to a socio-constructivist explanation, these children have usually experienced family contexts in which the processes of emotional socialization of coaching are lacking, particularly of negative emotions, and in which styles of dismissing or rejecting these emotions can often be found (Gottman et al., 1997; Shipman et al., 2007). The latter styles of emotional socialization do not allow moments of listening, understanding, and elaboration of emotions, and as such do not promote an adequate ability in children to understand their own and others’ emotions. According to a psychodynamic approach, instead, in abusive families the reflexive functions and the parental emotional containment are highly compromised, damaging the development of the child’s ability to mentalize (Fonagy and Fonagy, 2018).

In view of these data from the study I, the next step of the research, as previously illustrated, was to enhance, in the group of maltreated children, the perspective taking ability by means of a training already used in several previous studies (Mori and Cigala, 2019). Moreover, the aim was to verify the consequent development of adaptive behavior of these children in interactions with peers in the school context. The results of study II, although involving a small number of children, seem to be very encouraging and evidences the central role of the perspective taking ability in the promotion of adaptive behavior. In fact, participation in the training, alongside an increase in perspective taking ability, especially in its affective and cognitive components, has contributed to a significant increase in prosocial behavior, in particular helping and sharing. Since prosocial behavior was not the focus of the training conducted with the children, it is presumable that the increase in the frequency of these behaviors in the school context is connected to the children’s increased ability in perspective taking.

In line with the initial hypothesis of study II after the training, a decrease in aggressive behavior toward peers in the school context was observed. We believe that these results are particularly interesting both from a scientific and clinical point of view. From a scientific perspective, these results represent a further proof of the relationship between perspective taking and social adaptation in the developmental age. From a clinical point of view, these results allow us to outline possible interventions to promote and enhance the ability of children with histories of maltreatment to put themselves in another person’s shoes. An ability that, as the data show, could make it possible to break the vicious circle of aggression and allow children with a history of maltreatment to co-construct more positive interactions with their peers (Maughan and Cicchetti, 2002; Graham et al., 2010; Amédée et al., 2019). The statistical significance of the differences of aggressive behaviors from pre-test to post-test was not very high. Probably, in order to obtain a greater modification of aggressive behavior, a longer lasting training with a greater number of sessions would be necessary to consolidate the achievements.

The present studies have some important limitations: the small number of abused children involved, the lack of a control group (study II) and of a follow-up phase (study II), the impossibility to control some variables that could play a significant role, such as the type of maltreatment and finally the cross-sectional nature of the study that does not allow the identification of causal relationships between considered variables.

As regards the results obtained and the aforementioned limits, future research should replicate the studies on a larger sample and suggest interventions specifically focused on affective perspective taking, a dimension that has proven to be the most deficient. It would also be desirable to include a control group and to conduct a follow-up to assess the efficacy of intervention and the maintenance over time of the perspective taking ability acquired following the training. Moreover, other variables could be controlled in future studies, such as children’s emotion regulation, the socioeconomic status of maltreated children, and the type of maltreatment. It would also be interesting to realize longitudinal research designs that allow for the causal interpretations of the results. Finally, in future studies it could be of interest to carry out interventions that involve the entire community system, which includes the mothers of children as well as the stakeholders, so that daily life routines can become opportunities to take up different points of view.

Overall, the results of the studies have great practical implications as they allow us to identify in perspective taking, specifically the affective perspective taking, a key ability that can influence the social adaptation of preschoolers, increasing prosocial behaviors and decreasing aggressive behaviors among peers. In particular, for children with history of maltreatment the perspective taking ability represents a relevant protective factor in the development of positive social adjustment. Hence, it would be very useful in continuing a professional development path aimed at teachers and professionals who work daily with children, to disseminate and share this knowledge, because especially in everyday contexts, children can learn the perspective taking ability. Furthermore, from study II some relevant indications emerge regarding specific possible interventions applicable both in the school and community contexts, in order to promote the maltreated children’s capacity for affective, cognitive, and visual perspective taking.

## Data Availability Statement

The raw data supporting the conclusions of this article will be made available by the authors, without undue reservation.

## Ethics Statement

Ethical review and approval was not required for the study on human participants in accordance with the local legislation and institutional requirements. Written informed consent to participate in this study was provided by the participants’ legal guardian/next of kin.

## Author Contributions

AC and AM contributed to the design and implementation of the research, to the analysis of the results, and to the writing of the manuscript. All authors contributed to the article and approved the submitted version.

## Funding

This work was supported by Ministero dell’Istruzione, dell’Università e della Ricerca.

## Conflict of Interest

The authors declare that the research was conducted in the absence of any commercial or financial relationships that could be construed as a potential conflict of interest.

## Publisher’s Note

All claims expressed in this article are solely those of the authors and do not necessarily represent those of their affiliated organizations, or those of the publisher, the editors and the reviewers. Any product that may be evaluated in this article, or claim that may be made by its manufacturer, is not guaranteed or endorsed by the publisher.
